# Mesenchyme-free expansion and transplantation of adult alveolar progenitor cells: steps toward cell-based regenerative therapies

**DOI:** 10.1038/s41536-019-0080-9

**Published:** 2019-08-20

**Authors:** Aaron I. Weiner, Sergio R. Jackson, Gan Zhao, Kwaku K. Quansah, Joseph N. Farshchian, Katherine M. Neupauer, Elizabeth Q. Littauer, Andrew J. Paris, Derek C. Liberti, G. Scott Worthen, Edward E. Morrisey, Andrew E. Vaughan

**Affiliations:** 1 Department of Biomedical Sciences, School of Veterinary Medicine, University of Pennsylvania, Philadelphia, PA 19104 USA; 2 Institute for Regenerative Medicine, University of Pennsylvania, Philadelphia, PA 19104 USA; 3 Penn Center for Pulmonary Biology, University of Pennsylvania, Philadelphia, PA 19104 USA; 4 Department of Microbiology, Perelman School of Medicine, University of Pennsylvania, Philadelphia, PA 19104 USA; 5 Pulmonary, Allergy and Critical Care Division, Department of Medicine, Perelman School of Medicine, University of Pennsylvania, Philadelphia, PA 19104 USA; 6 Department of Cell and Developmental Biology, Perelman School of Medicine, University of Pennsylvania, Philadelphia, PA 19104 USA; 7 Penn Cardiovascular Institute, University of Pennsylvania, Philadelphia, PA 19104 USA; 8 Division of Neonatology, Department of Pediatrics, Perelman School of Medicine, University of Pennsylvania, Philadelphia, PA 19104 USA

**Keywords:** Adult stem cells, Regeneration

## Abstract

Alveolar type-2 (AT2) cells are necessary for the lung’s regenerative response to epithelial insults such as influenza. However, current methods to expand these cells rely on mesenchymal co-culture, complicating the possibility of transplantation following acute injury. Here we developed several mesenchyme-free culture conditions that promote growth of murine AT2 organoids. Transplanting dissociated AT2 organoids into influenza-infected mice demonstrated that organoids engraft and either proliferate as AT2 cells or unexpectedly adopt a basal cell-like fate associated with maladaptive regeneration. Alternatively, transplanted primary AT2 cells also robustly engraft, maintaining their AT2 lineage while replenishing the alveolar type-1 (AT1) cell population in the epithelium. Importantly, pulse oximetry revealed significant increase in blood-oxygen saturation in primary AT2 recipients, indicating that transplanted cells also confer increased pulmonary function after influenza. We further demonstrated that both acid installation and bleomycin injury models are also amenable to AT2 transplantation. These studies provide additional methods to study AT2 progenitor potential, while serving as proof-of-principle for adoptive transfer of alveolar progenitors in potential therapeutic applications.

## Introduction

The lung is able to regenerate following severe injury such as influenza due to resident alveolar type-2 (AT2) cells within the alveoli. AT2 cells can self-renew and differentiate into alveolar type-1 (AT1) cells, thus producing essential surfactant and providing a source of oxygen-exchanging epithelial AT1 cells.^1,2^ However, this response is imperfect and the lung also exhibits aberrant tissue remodeling that results in the appearance of ectopic cell types in the alveoli, especially bronchiolized epithelium^3^ containing solitary chemosensory/tuft cells.^4^ This dysplastic tissue arises largely due to expansion and occupation of the alveoli by p63^+^ Krt5^+^ cells derived from migratory basal-like “lineage-negative” cell populations.^3,5–7^ Initially described in mice, there is increasing evidence that this dysplastic response also occurs in humans and may impair long-term functional recovery.^8–10^

Here we describe two methods to promote lung regeneration after influenza infection: transplantation of AT2 organoids grown in defined culture conditions and transplantation of primary AT2 cells (Supplementary Fig. S1). Established methods for AT2 organoids require co-culture with lung mesenchyme,^2,11^ of which the mesenchymal alveolar niche cell (MANC) subpopulation primarily supports AT2 self-renewal in vivo and in vitro.^12^ Despite its benefits for ex vivo AT2 maintenance, lung mesenchyme co-culture is an undesirable system for subsequent transplantation, as separation of the intermingled cell types in vitro is technically challenging and transplantation of mesenchyme can exacerbate organ repair and fibrosis following injury.^13–15^ Specifically, fibroblast-like cells present in mesenchymal co-culture conditions are predominant sources of, and responders to, transforming growth factor-β and other fibrosis-inducing growth factors and cytokines.^16–19^ In addition, the pro-AT2 signals secreted by the lung mesenchyme vary in concentration and composition, making lung mesenchyme co-culture disadvantageous for our goal of defining precise ex vivo AT2 growth conditions. The AT2 culture methods described here allow for well-defined in vitro expansion of AT2 organoids, while abrogating the need for supporting mesenchymal cells, permitting organoid transplantation. Primary AT2 cells were also transplanted, responding similarly to native AT2 progenitors by proliferating and differentiating into AT1 cells. Importantly, primary AT2 transplant measurably improved pulmonary function as indicated by faster recovery of blood-oxygen saturation. This report provides techniques that can be leveraged into future therapeutic approaches in lung regeneration.

## Results

**Fig. 1:**
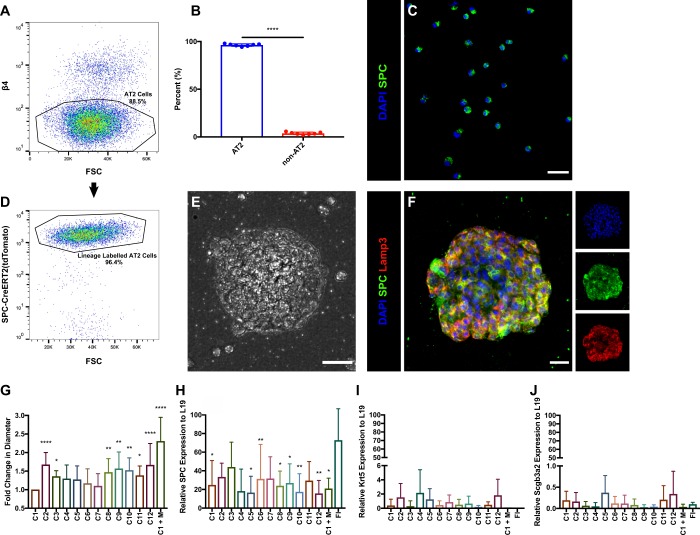
Mesenchyme-free culture conditions generate healthy AT2 organoids. **a** FACS isolation of AT2 cells by gating on β4^−^ lung epithelial cells. **b** AT2-sorted purity quantification by manual cell count of cytospins yields a 96.25 ± 0.47% pure population. *n* = 7 from 7 independent experiments, mean ± SEM, ****p < 0.0001 by two-tailed Welch’s *t*-test. **c** Representative immunofluoresence image of an AT2 cytospin used for purity checks. Scale bar = 25 μm. **d** Validation of gating scheme and purity by subgating β4^−^ epithelial cells from a tamoxifen-administered *SPC-CreERT2(tdTomato)* reporter mouse. 96.4% of β4^−^ cells were lineage-traced, similar to cytospin purity quantification. **e**, **f** Representative bright-field max projection and immunofluorescence images of cytospun AT2 organoids grown in C2 for 9 days. Scale bar = 25 μm. **g** Change in organoid diameter between culture conditions, normalized to the average diameter of C1 organoids. Significance tests are relative to C1. **h**–**j** qPCR shows that many culture conditions maintain high SPC expression (**h**), whereas expression of Krt5 (**i**) and Scgb3a2 (**j**) remain low across all conditions. Significance tests are relative to freshly isolated (FI) AT2 expression of corresponding genes. Data for **g**–**j** are based on *n* ≥ 3 for all conditions from at least 15 independent experiments and error bars represent SD

We initially asked whether utilizing growth factors and modulators of signaling pathways involved in lung development might allow for culture of pure AT2 cells, removing the need for mesenchymal support. AT2 cells were flow sorted using an established gating scheme^20^ (Fig. 1a and Supplementary Fig. 2). Cytospins validated this strategy, yielding a 96.25 ± 0.47% pure AT2 population (Fig. 1b, c), which we additionally confirmed by sorting from a tamoxifen-treated *SPC-CreERT2(tdTomato)* reporter mouse in which 96.4% of gated cells were lineage-labeled AT2s (Fig. 1d). Capitalizing on incorporation of developmental signals such as Wnt, fibroblast growth factor (FGF), and bone morphogenetic protein (BMP) signaling, we modified existing culture conditions^3,6^ to promote mesenchyme-free growth of purified AT2 cells. Eleven culture conditions were tested (C1–C11), in addition to a serum-free condition containing all growth factors (C12) and a mesenchymal co-culture condition (C1 + M) (Table 1). The lung mesenchyme population for C1 + M was isolated by a CD45^−^ PECAM^−^ EpCAM^−^ sorting strategy (Supplementary Fig. 3). This population consisted largely of Pdgfrα^+^ (~53% of sorted lung mesenchyme) cells, enriched in MANCs, and Wnt2^+^ (~6% of sorted lung mesenchyme) cells, as well as αSMA^+^ airway smooth muscle cells and/or myofibroblasts (~4% of sorted lung mesenchyme) (Supplementary Fig. 3). AT2 cells grew into spherical organoids after 13 days in culture (Fig. 1e). Immunostaining displayed expression of canonical AT2 markers such as surfactant protein C (SPC) and Lamp3 (Fig. 1f), and quantitative PCR (qPCR) confirmed that most conditions maintained expression levels of SPC comparable to freshly isolated (FI) AT2 cells (Fig. 1h). However, some conditions showed slightly higher expression of Scgb3a2, an airway cell marker, and cytokeratin 5 (Krt5), an indicator of lung dysplasia (Fig. 1i, j). Diameter was used to assess proliferative ability and overall health (Fig. 1g). In accordance with established methods,^2^ mesenchymal co-culture generated the largest relative organoids. Predictably, conditions containing all or most growth factors generated the largest spheroids (Supplementary Fig. 4a–c).

**Table 1:** Contents and concentration of growth factors and cell culture supplements used in each AT2 organoid culture condition

	Final working concentration	C1	C2	C3	C4	C5	C6	C7	C8	C9	C10	C11	C12	C1+M
SAGM	–	+	+	+	+	+	+	+	+	+	+	+	–	+
ADMEM/F12	–	–	–	–	–	–	–	–	–	–	–	–	+	–
KGF	20 ng/mL	+	+	+	+	+	+	+	+	+	+	+	+	+
EGF	50 ng/mL	+	+	+	+	+	+	+	+	+	+	+	+	+
A83-01	1 μM	–	+	+	+	+	+	+	+	+	+	+	+	–
Rspo1	200 ng/mL	–	+	+	+	+	+	–	+	+	+	–	+	–
Wnt3a	100 ng/mL	–	+	+	+	+	–	–	+	+	–	+	+	–
Y-27532	5 μM	–	+	+	+	–	–	–	+	–	+	+	+	–
Noggin	100 ng/mL	–	+	+	–	–	–	–	–	+	+	+	+	–
FGF10	100 ng/mL	–	+	–	–	–	–	–	+	+	+	+	+	–
B27	1×	–	–	–	–	–	–	–	–	–	–	–	+	–
N2	1×	–	–	–	–	–	–	–	–	–	–	–	+	–
HEPES	10 mM	–	–	–	–	–	–	–	–	–	–	–	+	–
Gentamycin	1×	–	–	–	–	–	–	-	–	–	–	–	+	–
P/S	1×	–	–	–	–	–	–	–	–	–	–	–	+	–
Lung mesenchyme	26,000 cells	–	–	–	–	–	-	–	–	–	–	–	–	+

**Fig. 2:**
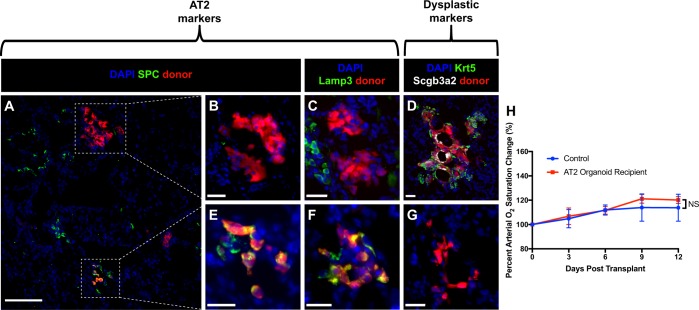
AT2 organoids display a bipotential fate upon post-injury transplantation. **a** Immunofluorescence of injured recipient lungs receiving 50,000–100,000 AT2 organoid cells reveals proximate engraftments with differing cell fates. Scale bar = 100 μm. **b**–**g** Representative images of AT2 organoid engraftments. Some AT2 organoid engraftments adopt a maladaptive fate, exclusively expressing the dysplastic markers Krt5 and Scgb3a2 (**d**) as opposed to markers of their original AT2 fate (SPC and Lamp3) (**b**, **c**). Other engraftments remain as AT2 cells and maintain AT2 marker expression (**e**, **f**) without expressing dysplastic markers (**g**). Immunostaining expression patterns were observed in five mice from three independent experiments. Scale bars = 25 μm. **h** Pulse oximetry readings were taken from 100,000 AT2 organoid cell-transplanted and mock-transplanted mice over the course of 12 days post transplant. No significant increase in %O_2_ was detected. Data for **h** is based on control *n* = 7 mice, transplanted *n* = 3 mice from three independent experiments, and error bars represent SD. NS ≥ 0.05, *p < 0.05, **p < 0.01, ***p < 0.001, ****p < 0.0001 by ordinary one-way ANOVA

Utilizing two promising conditions, C2 and C12, AT2 organoids were grown, dissociated, and transplanted into influenza-injured recipient mice 11 days post infection (DPI) as previously described.^3^ Immunostaining of recipient lungs 13 days post transplant (DPT, 24 DPI) revealed that engraftments could adopt two distinct fates (Fig. 2a): maintenance of the AT2 lineage (SPC and Lamp3 expression) (Fig. 2e, f) or, surprisingly, expression of markers of dysplastic regeneration (Scgb3a2 and Krt5) (Fig. 2d) despite low expression of these genes in culture. These expression patterns were mutually exclusive; dysplastic engraftments never expressed AT2 markers (Fig. 2b, c) and vice versa (Fig. 2g). Pulse oximetry of organoid transplant recipients was not significantly different than that of mock-transplanted mice (Fig. 2h). Together, these data indicate that although sorted AT2 cells can be expanded in mesenchyme-free culture conditions without disrupting their AT2 fate, these organoids can spontaneously adopt a dysplastic fate in vivo.

**Fig. 3:**
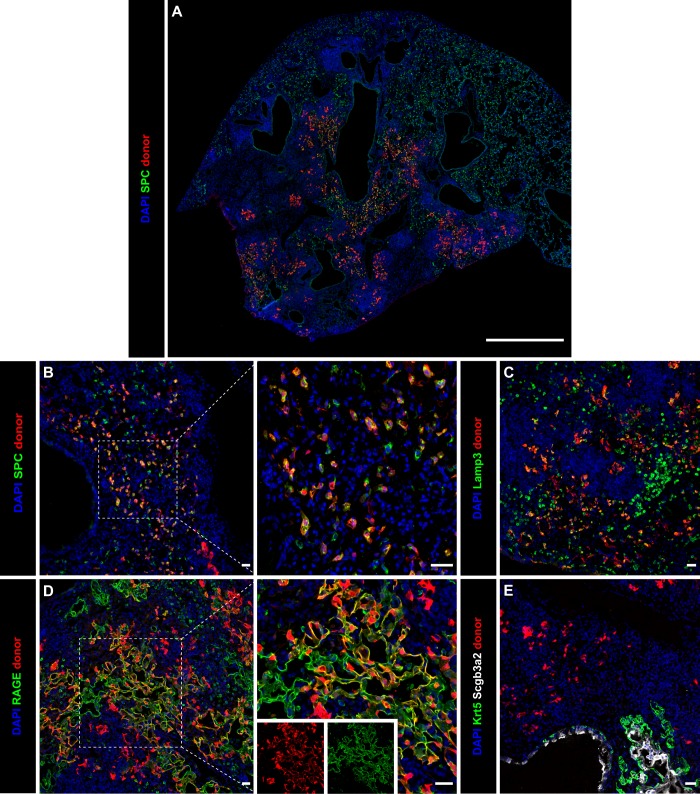
Primary adult AT2 cell engraftments exclusively maintain an AT2 cell fate in vivo and contribute to re-epithelialization of the injured lung. **a** Whole-lobe immunostain of a primary AT2 transplant recipient, showing extent of engraftment and proliferation throughout injured lobes. Scale bar = 1000 μm. **b**–**g** Representative immunostains of AT2, AT1, and dysplastic markers demonstrating the fate of engrafted primary AT2 cells. Transplanted primary AT2 cells either expand as AT2 cells, evidenced by SPC (**b**) and Lamp3 (**c**) expression, or differentiate into AT1 cells expressing RAGE (**d**). Engraftments expand into zones exhibiting dysplasia but never express the dysplastic markers Krt5 and Scgb3a2 (**e**). Insets in **b** and **d** are of dashed boxes in larger image to highlight SPC and RAGE expression. Immunostaining expression patterns were observed in six mice from two independent experiments. Scale bars = 25 μm

**Fig. 4:**
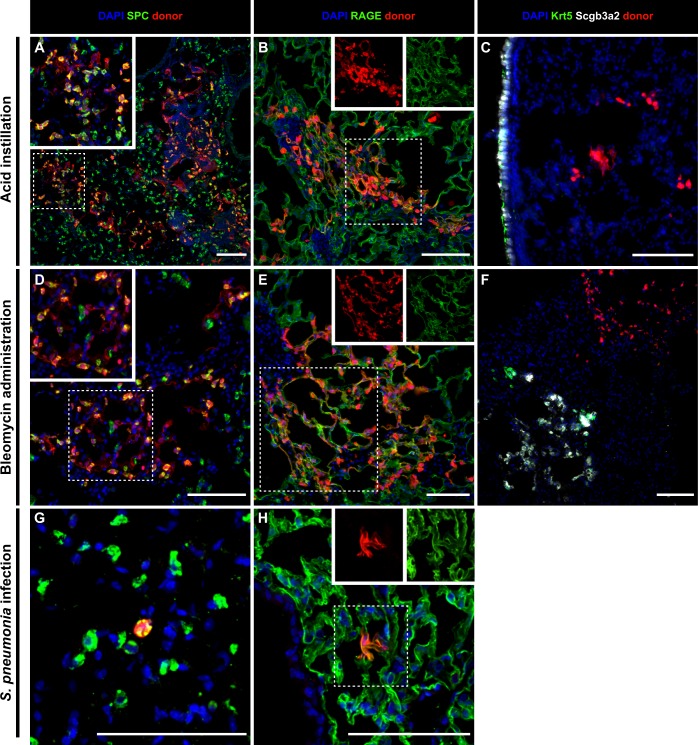
Primary AT2 transplants into alternative injury models. Primary AT2 cells were transplanted into acid- (**a**–**c**), bleomycin- (**d**–**f**), and *Sp*-injured (**g**, **h**) recipients. Transplanted AT2s engraft and expand exclusively as AT2s (**a**, **d**, **g**) or AT1s (**b**, **e**, **h**) in all injury models and never contributed to dysplasia (**c**, **f**). Magnified sections and individual channels are derived from dashed boxes to highlight individual cell/marker expression. Scale bars = 100 μm

Given the unexpected expression of dysplastic markers upon organoid transplant, we reasoned that primary AT2 cells, which never experience in vitro conditions, may retain more appropriate lineage restriction upon transplant. Indeed, primary AT2 cell transplants robustly engraft and expand throughout a significant portion of influenza-injured lobes (Fig. 3a). Immunostaining of recipient lungs 13 DPT revealed that all engrafted primary AT2 cells either remain as AT2 cells (Fig. 3b, c) or differentiate into AT1 cells (Fig. 3d and Supplementary Fig. 5a, b). In contrast with organoid engraftments (Supplementary Fig. 6c), none of the primary cell engraftments express Scgb3a2 or Krt5 (Fig. 3e and Supplementary Fig. 6a, b). Primary AT2 cells were also transplanted into an array of other injury models to determine whether the extent or type of damage influenced the ability of primary AT2s to engraft. Robust engraftments were observed in 4/4 acid-injured and 4/4 bleomycin-injured mice at 13 DPT (14 days post acid instillation and 23 days post bleomycin administration, respectively). These engraftments exclusively contributed to AT2 and AT1 regeneration similarly to influenza-injured recipients and never exhibited dysplastic regeneration markers (Fig. 4a–f). Interestingly, primary AT2 transplants into *Streptococcus pneumonia* (*Sp*)-infected recipients resulted in only rare engraftments consisting of 1–3 cells (Fig. 4g, h). These results suggest that primary AT2 cells are capable of engrafting into a diverse array of pulmonary injury models, properly maintain their fate and in vivo function, and aid in re-epithelializing the damaged lung.

**Fig. 5:**
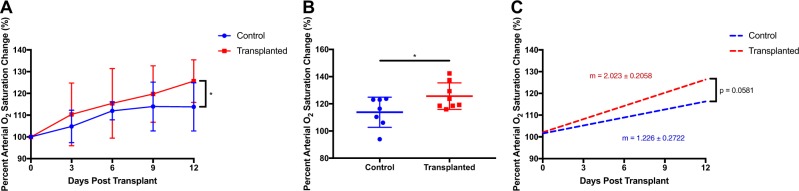
Primary AT2 transplants improve pulmonary function post flu. **a**, **b** Pulse oximetry readings were taken from primary AT2- and mock-transplanted mice over the course of 12 days post transplant. A significant increase in percent change of %O_2_ was detected at day 12 post transplant. **c** Linear regression of pulse oximetry data reveals an increasing trend in the slope of recovery of lung function in primary AT2 recipient mice. Data for **f**–**h** is based on control *n* = 7 mice, transplanted *n* = 8 mice from four independent experiments. Error bars represent SD. *p < 0.05 by two-way ANOVA. Linear regression slope *p*-value based on ANCOVA

Strikingly, pulse oximetry revealed that blood-oxygen saturation was significantly higher 12 DPT (23 DPI) in influenza-injured recipient mice compared with that of mock-transplanted controls (*p* = 0.0315) (Fig. 5a, b). Linear regression analysis also indicated that the slope of blood-oxygen saturation change over time for transplante recipient mice was very nearly statistically significantly higher (*p* = 0.0581) than that of control mice (Fig. 5c), indicating that primary cell-transplanted mice recover ~65% faster than controls. We conclude that primary AT2 cells are capable of engrafting into injured murine lungs, serving their role as stem cells by partially replenishing the AT1 and AT2 cell populations and aiding in restoration of pulmonary function.

## Discussion

This report provides methods to expand and transplant AT2 cells. Factors that promote branching morphogenesis and AT2 expansion such as FGF10, KGF,^21^ and Wnt^22^ proved sufficient to expand AT2 organoids without mesenchymal co-culture, yielding large organoids with high SPC expression. Interestingly, we saw a stepwise decline in organoid diameter with each growth factor removed from conditions C2 to C7, suggesting an additive effect of each factor. This approach failed to reveal a single growth factor that was absolutely required for maintaining either SPC expression or proliferative ability. Organoid diameter in mesenchyme-free culture conditions approached but never matched that of co-culture, suggesting that additional factors will be necessary to maximize organoid size, or that some growth signals are derived via direct cell–cell interactions. Similarly, organoid SPC levels did not reach those of uncultured AT2 cells, suggesting that further optimization is possible.

AT2 organoids engraft in vivo without the use of complex delivery or expansion systems, many retaining their AT2 fate. Despite this, organoids sometimes adopt a dysplastic fate upon engraftment, a surprising finding given that definitive lineage tracing has demonstrated that Krt5^+^ cells are never derived from endogenous AT2 cells in vivo.^3^ Considering negligible expression of dysplastic markers in vitro (Fig. 1i, j), differentiation into maladaptive fates could result from unintentional “priming” in vitro that manifests in vivo. Transplantation of AT2 organoids did not appear to increase the oxygen-exchange capability of injured recipient mice, likely to be due to the contribution of engraftments to non-oxygen-exchanging dysplastic tissue. Further elucidation of the transcriptional changes that occur in AT2 organoids and the signals encountered by transplanted organoids in the influenza-injured microenvironment/engraftment niche should provide insight into how to further optimize organoid transplant.

In contrast to organoid transplants, primary cell engraftments either expande as AT2 cells or differentiate into AT1 cells, indicating that transplanted adult AT2s have the capability to robustly differentiate into AT1 cells in vivo after a wide array of pulmonary insults, analogously to their native post-flu counterparts. Retention of solely alveolar markers in primary cell engraftments further indicates that in vitro culturing is likely responsible for dysplastic organoid differentiation. Interestingly, only small clusters of a few AT2s from primary AT2 transplants were found in the lungs of *Sp*-infected recipient mice, in stark contrast to the widespread donor AT2 expansion found in other injury model recipients. This could be due to the presence of active infectious bacteria in the post-*Sp* lung at the time of transplant,^23^ whereas the other injury models used had either cleared the infection by the time of transplant or did not use infectious agents. Further studies will be needed to optimize the timing of adoptive AT2 transfer and to examine the possibility of transplant during bouts of active infection.

Pulse oximetry confirmed that transplanted primary AT2s assist in restoring the oxygen-exchange capacity of the epithelium, improving pulmonary function. The upward trend in oxygen saturation becomes statistically significant at 12 DPT in transplant recipients, demonstrating that primary AT2 transplantation confers a true restorative advantage at a relatively early time point in recovery. It remains to be determined whether functional benefits of cell transplant are mediated mostly by restoration of gas-exchanging AT1 cells, supplementation of surfactant production, or, likely, a combination of both. Long-term studies will be necessary to assess the longevity of transplanted primary cells and determine the ultimate extent to which they restore pulmonary function.

Orthotopic transplantation of adult progenitor cells and induced pluripotent stem cells (iPSCs) has been employed to restore physiological function in other organ systems. Transplantation of adult human hepatocytes and hepatic iPSC-derived organ buds into a mouse model of liver failure rescues this lethal genetic phenotype, with active in vivo contribution of the engrafted cells to albumin synthesis and drug metabolism.^24,25^ Similar experiments have been performed in the mammary gland, in which transplantation of a single mammary stem cell into cleared murine mammary fat pads is able to fully reconstitute a functional gland with milk-producing capability.^26^ Fetal murine enteric progenitors^27^ and human colon organoids^28^ are also capable of regenerating full intestinal crypts and villi following dextran sulfate sodium-induced ulcerative colitis and de-epithelialization, respectively, although the extent to which these engraftments contribute to intestinal function has yet to be determined. Few studies have attempted epithelial progenitor cell transplant in the lung and fewer yet have assessed the functional outcomes of cell transplant. Transplantation of airway-derived progenitor cells, as opposed to AT2s, into the alveoli of influenza-injured mice has been performed by different groups,^3,5^ although studies of the resulting engraftments primarily focus on their in vivo cell-fate outcomes. In a creative approach, one group recently transplanted AT2 cells that underwent “interrupted reprogramming” via transient expression of the pluripotency factors into bleomycin-injured mice, improving metrics of tissue resistance and compliance, while seemingly helping to mitigate fibrosis.^29^ Our study similarly finds that orthotopic transplantation of mature AT2 cells can directly contribute to restoration of the most critical pulmonary function, gas exchange.

Although further optimization of culture conditions to retain normal cell-fate restriction is still required, transplantation of primary AT2 cells demonstrated significant improvements in lung function recovery. The strict retention of alveolar fate and expansion in harsh injury environments of primary AT2s highlights the robustness of this progenitor population and underscores its utility as a source of progenitors for syngeneic transplant of alveolar epithelial cells following pulmonary insults. This work begins to address the need for mesenchyme-free culture conditions that permit transplantation of lineage-restricted and expandable AT2 organoids, while highlighting the feasibility of adoptive transfer of alveolar progenitors for future therapies.

## Methods

### Animals and treatment

8- to 10-week-old mice were used for all experiments with males and females in roughly equal proportions. Experimenters were not blinded to mouse age or sexes. *Ai14-tdTomato* (*Gt(ROSA)26Sor*^*tm14(CAG-tdTomato)Hze*^),^30^ *dsRed* (Tg(CAG-DsRed*MST)1Nagy),^31^ *UBI-GFP* (*Tg(UBC-GFP)30Scha*),^32^ *R26R-eYFP* (*Gt(ROSA)26Sor*^*tm1(EYFP)Cos*^),^33^ *SPC-GFP* (*Tg(Sftpc,-EGFP)1Dobb*),^34^ *SPC-CreERT2* (*Sftpc*^*tm1(cre/ERT2,rtTA)Hap*^),^20^ *Pdgfrα-GFP (Pdgfra*^*tm11(EGFP)Sor*^*)*,^35^ and *Wnt2-CreERT2*^36^ mice have been previously described. All studies were approved by the University of Pennsylvania’s Institutional Animal Care and Use Committees, protocol 806262, and followed all NIH Office of Laboratory Animal Welfare regulations.

### Influenza infection

Control and infected mice were first anesthetized using 3.5% isoflurane in 100% O_2_ via an anesthesia vaporizer system. Mice were intranasally administered 40–70 U TCID50 of influenza A/H1N1/Puerto Rico/8/34 (PR8) by pipetting 30 μL of virus dissolved in phosphate-buffered saline (PBS) onto the nostrils of anesthetized mice in visually confirmed agonal breathing. Control mice were administered 30 μL PBS using the same method. Only infected mice that lost ≥15% of their starting body weight by 11 DPI were considered to be adequately infected and were used for all experiments involving influenza infection. Mouse weights were tracked by measuring at days 0, 3, 6, 9, 14, 17, 20, and 23 post infection. Cells were transplanted at day 11 post infection.

### Acid injury

Mice were anesthetized by intraperitoneal injections of 100 μL ketamine/xylazine (100 mg/kg). Sedated mice were intubated with a 20 G angiocatheter (BD) as previously described.^37^ Mice were then placed in the right lateral recumbent position and a polyethylene 10 (PE10) catheter (Clay Adams) was directed into the right main stem bronchus, while pressure was applied to the left lung. Injury was induced by instilling 2 μL/g of osmotically balanced 0.1 N HCl into the right lung (maximum dose = 50 μL) through the PE10 catheter as previously described.^38^ Cells were transplanted at day 1 post acid administration.

### Bleomycin administration

For bleomycin-induced lung injury, we utilized intranasal administration as described for influenza infection with the following modifications. Mice received between 1.5 and 2.25 mg/g body weight bleomycin sulfate (13877-10, Cayman Chemicals) in a total volume of 50 μL PBS. Given the inherent variability of bleomycin injury, this range of doses was used to ensure a cohort of mice that was sufficiently injured (lost ≥10% body weight) but survived the injury. Cells were transplanted at day 10 post bleomycin administration.

### *S. pneumonia* infection

*Sp* strain TIGR4 (serotype 4) was grown in tryptic soy broth for 2 hours as previously described.^39^ Mice to be infected were anesthetized by intraperitoneal injections of 100 μL ketamine/xylazine (100 mg/kg) and inoculated intranasally with 30 μL of 5.3 × 10^7^ CFU *Sp* suspension. Cells were transplanted 2 days post *Sp* administration.

### Organoid culture

CD45^−^ EPCAM^+^ β4^−^ AT2 cells used for organoid culture experiments were fluorescence-activated cell (FACS) sorted from *C57BL6*, *SPC-GFP*, *UBI-GFP*, or *dsRed* mice. For each growth condition, 80,000 cells were seeded onto 90 μL of Matrigel (BD) in a Corning^®^ 96-well clear polystyrene flat-bottom microplate (Millipore Sigma). Organoid culture reagents used are as follows and each culture condition combination in addition to final in-solution concentrations of growth factors can be found in Table 1: 5% charcoal-treated fetal bovine serum (FBS) (Life Technologies), penicillin–streptomycin (P/S) solution (ATCC), SABM^TM^ Basal Medium (Lonza) + SAGM^TM^ SingleQuots^TM^ Supplement Pack (Lonza) excluding hydrocortisone, gentamycin solution (Millipore Sigma), A83-01 (Millipore Sigma), recombinant murine Wnt3a (Peprotech), recombinant murine Noggin (Peprotech), recombinant human FGF10 (Peprotech), recombinant human KGF (FGF7, Peprotech), recombinant murine EGF (Peprotech), recombinant human Rspondin-1 (Peprotech), Y-27632 dihydrochloride (Stemcell Technologies), B27^TM^ supplement (50 × , Thermo Fisher Scientific), GlutaMAX^TM^ supplement (100 × , Thermo Fisher Scientific), HEPES buffer solution (1 M, Thermo Fisher Scientific), and N2 supplement (100 × , Thermo Fisher Scientific). Organoids cultured in all conditions were grown in a 37 °C incubator for 13 days without passaging and with media changes on day 5 and day 9 before collecting for RNA or transplant.

### Organoid diameter quantification

Organoids grown from all mice listed in “Organoid culture” were used for diameter quantification. Tilescans of organoid-containing wells were imaged using the bright-field setting on a Leica DMi8 Microscope using a Leica DFC9000 sCMOS camera and Leica Application Suite X (LASX) software. Organoids were always grown in at least technical duplicates and all technical replicate wells were imaged and quantified. Diameter measurement was performed by using the built-in “Draw scalebar” function on LASX. Ten to 50 organoids were measured and averaged in each technical replicate, and technical replicates were averaged for a final biological replicate, consisting of >30 organoids measured across all technical replicates. The resultant diameter from each biological replicate was then normalized to the average diameter of C1 organoids, which were grown as a control in at least one technical replicate for all experiments. Reported average diameter for each condition comes from at least *n* = 3 biological replicates.

### Organoid and primary cell transplants

*dsRed* mice bred to a *C57BL6* background were used to grow donor organoids for organoid transplant experiments. Organoid transplant recipients received 20,000– 150,000 AT2 organoid cells grown in C2 and C12 for 13 days with media changes on days 5 and 9. *UBI-GFP*, *SPC-GFP*, or *SPC-CreERT2(Ai14-tdTomato)* mice bred to a *C57BL6* background were used as donors for transplant experiments. *SPC-CreERT2(Ai14-tdTomato)* mice were given three doses of 0.25 mg/g body weight tamoxifen dissolved in 50 μL corn oil every other day for 6 days. Mice given tamoxifen were killed for FACS 1 week following the final dose. Primary AT2 recipients received 900,000 CD45^−^ EPCAM^+^ β4^−^ AT2 cells. All recipients of both organoid and primary cell transplants were *C57BL6*. Recipient mice anesthetized with 3.5% isoflurane in 100% O_2_ via an anesthesia vaporizer system were intranasally administered cells by pipetting single-cell suspension in PBS + 1% P/S onto the nostrils of anesthetized mice in visually confirmed agonal breathing.

### Collecting organoids for RNA and transplants

Organoids were treated with 15 U/mL dispase II (Roche) in Hank’s balanced salt solution (HBSS) for 30 minutes, rinsed with 2 mM EDTA (Thermo Fisher Scientific), incubated in 2 mM EDTA for 5 min at 37 °C, mechanically dissociated by pipetting 50–100 times with a p200, and pelleted at 550 × *g* for 5 minutes at 4 °C. Cell pellets to be used for RNA were frozen by first aspirating supernatant followed by direct placement in a −80 °C freezer. Cell pellets to be used for organoid transplant had their supernatant aspirated and were resuspended in 30 μL PBS + 1% P/S per number of cells to be transplanted. Resuspended organoid transplant cells were kept on ice between final resuspension and transplantation.

### Preparation of primary AT2 cells for transplant

Directly after sorting, primary AT2 cells were pelleted at 550 × *g* for 5 minutes at 4 °C. After aspirating the supernatant, the cell pellet was resuspended in 30 μL PBS + 1% P/S per 900,000 cells to be transplanted. Resuspended primary AT2 cells were kept on ice between final resuspension and transplantation.

### Pulse oximetry

Repeated measurements of peripheral oxygen saturation (SpO_2_) were taken using a MouseOx Plus Rat & Mouse Pulse Oximeter and a MouseOx small collar sensor (Starr Life Sciences Corp.). Mice were shaven around the neck and shoulders where the collar sensor sits. Recordings were taken using MouseOx Premium Software (Starr Life Sciences Corp., Oakmont, PA, USA). Measurements were taken continuously for > 3 minutes at a measurement rate of 15 Hz. Measurements were imported into Microsoft Excel and all readings with a non-zero Error Code were filtered out. The average of these error-free readings was used to calculate the SpO_2_ reading for each mouse for each given time point. SpO_2_ measurements were normalized to pretreatment SpO_2_ values at day 0.

### Lung tissue preparation for immunostaining

Following sacrifice via isoflurane overdose, lungs were inflated at a constant pressure of 25 cm H_2_O with 3.2% paraformaldehyde (PFA) for 30 minutes followed by incubation in 3.2% PFA for another 30 minutes at room temperature. Fixed lungs were then washed in multiple PBS washes over the course of 1 hour at room temperature, followed by an overnight incubation in 30% sucrose shaking at 4 °C, and then a 2 hour incubation in 15% sucrose 50% OCT compound (Fisher HealthCare) at room temperature. Finally, fixed lungs were embedded in OCT by flash freezing with dry ice and ethanol.

### Organoid preparation for cytospins

Organoids were collected by first washing each organoid well with PBS, then scraping the Matrigel plug and organoids into a 15 mL tube using a p20 pipet tip. Each well was washed once more with PBS and all washes were collected in their respective tubes. Each tube was topped off with 10 mL cold PBS and the organoids in suspension were gently pipetted up to ten times with a p1000 to break up the Matrigel. Organoids were pelleted at 200 × *g* for 5 minutes at 4 °C, resuspended in 10 mL cold PBS, and shaken at room temperature on ice for 30 min. Organoids were once more gently pipetted up to ten times to break up the remaining Matrigel before pelleting organoids again at 200 × *g* for 5 minutes at 4 °C. Organoids were resuspended in 10 mL cold 3.2% PFA and shaken at room temperature on ice for 30 min. Organoids were pelleted once more at 200 × *g* for 5 minutes at 4 °C.

### Cytospins

Sorted cells were pelleted at 550 × *g* and organoids at 200 × *g* for 5 minutes at 4 °C before resuspension in 200 μL 3.2% PFA per number of slides to be cytospun. 200 μL of resuspended cells or organoids was loaded into cytospin chambers. Cells were spun at 75 r.p.m. for 4 minutes and organoids were spun at 200 rpm for 5 minutes on a Cytospin 2 (Shandon).

### Immunostaining

Seven-micrometer sections were cut on a Leica CM3050 S Research Cryostat (Leica Biosystems). Tissue sections and cytospins were further fixed for 5 minutes in 3.2% PFA, rinsed three times with PBS, and blocked in blocking solution (PBS + 1% bovine serum albumin (Affymetrix) + 5% normal donkey serum (Jackson Immuno Research) + 0.1% Triton X-100 (Millipore Sigma)+ 0.02% sodium azide (Millipore Sigma)) for > 30 minutes. Slides were incubated in primary antibodies (listed below) in blocking solution overnight at 4 °C. Slides were then washed three times with PBS + 0.1% Tween-20 (Millipore Sigma) and subsequently incubated with secondary antibodies (listed below) for > 2 h at room temperature. Slides were then washed once more with PBS + 0.1% Tween-20 prior to incubation in 1 μM DAPI (Life Technologies) for 5 minutes, rinsed with PBS, and mounted with either Prolong Gold (Life Sciences) or Fluoroshield (Millipore Sigma). The following primary antibodies were used: rabbit anti-SPC (1:2000, Millipore), rat anti-Lamp3 (1:500, Novus, clone 1010E1.01), rat anti-RAGE (1:500, R&D, clone 175410), rabbit anti-Aqp5 (1:100, Abcam, clone EPR3747), Syrian hamster anti-Pdpn (1:100, DSHB, clone 8.1.1), rabbit anti-Krt5 (1:1000, BioLegend, clone Poly19055), chicken anti-Krt5 (1:500, BioLegend, clone Poly9059), goat anti-Scgb3a2 (1:200, R&D, clone AF3465), and sheep anti-eGFP (1:500, Invitrogen, 10396164). The following secondary antibodies were used: Alexa Fluor™ 488-conjugated donkey anti-sheep (1:1000, Thermo Fisher Scientific), Alexa Fluor™ 488-conjugated donkey anti-rabbit (1:1000, Thermo Fisher Scientific), Alexa Fluor™ 488 donkey anti-rat (1:1000, Thermo Fisher Scientific), donkey anti-chicken Alexa Fluor™ 488 (1:500, Jackson Immuno Research), fluorescein isothiocyanate-conjugated goat anti-chicken (1:1000, BioLegend, Poly24108), Alexa Fluor™ 568-conjugated donkey anti-rabbit (1:1000, Thermo Fisher Scientific), Alexa Fluor™ 568-conjugated donkey anti-goat (1:1000, Thermo Fisher Scientific), Alexa Fluor™ 647-conjugated donkey anti-rabbit (1:1000, Thermo Fisher Scientific), Alexa Fluor™ 647-conjugated donkey anti-goat (1:1000, Thermo Fisher Scientific), Alexa Fluor™ 647-conjugated chicken anti-rat (1:1000, Thermo Fisher Scientific).

### Fluorescence-activated cell sorting

Lung cells were isolated by first inflating lungs with 15 U/mL dispase II in HBSS (Thermo Fisher Scientific), tying off the trachea, and cutting lobes away from the main stem bronchi. Lobes were then incubated in dispase for 45 minutes shaking at room temperature and mechanically dissociated by pipetting in sort buffer (SB; Dulbecco’s modified Eagle’s medium (DMEM) (Thermo Fisher Scientific) + 2% cosmic calf serum (CC; Thermo Fisher Scientific) + 1% P/S). After pelleting at 550 × *g* for 5 minutes at 4 °C, whole-lung suspension was treated with Red Blood Cell Lysis Buffer (Millipore Sigma) for 5 minutes, pelleted, and resuspended in SB + 1:1000 DNase I (Millipore Sigma) for a 45 minutes recovery period shaking at 37 °C. Whole-lung suspension was then repelleted and resuspended in SB + 1:50 TruStain FcX™ (anti-mouse CD16/32) Antibody (BioLegend) for a 10 minutes blocking period at 37 °C. For sorts from *C57BL6* mice, AT2 cells were collected using allophycocyanin (APC)/Cy7-conjugated rat anti-mouse CD45 antibody (1:200, BioLegend, 30-F11), Alexa Fluor^®^ 488-conjugated rat anti-mouse CD326 (EpCAM) antibody (1:200, BioLegend, G8.8), and phycoerythrin (PE)-conjugated rat anti-mouse CD104 (integrin β4) antibody (1:100, BioLegend, 346-11A). For sorts from mice expressing a *GFP* fluorophore, AT2 cells were collected using the *C57BL6* sort scheme above but with PE-conjugated rat anti-mouse EpCAM (1:200, BioLegend, G8.8) and APC-conjugated rat anti-mouse integrin β4 antibody (1:100, BioLegend, 346-11A) instead of the respective EpCAM and integrin β4 antibodies listed above. For sorts from mice expressing a *RFP* fluorophore, AT2 cells were collected using the *C57BL6* sort scheme above but with APC-conjugated rat anti-mouse integrin β4 antibody instead of the respective integrin β4 antibody listed above. Lung mesenchyme collected from all aforementioned mice was collected by adding APC/Fire™ 750-conjugated rat anti-mouse CD31 (PECAM) (1:200, BioLegend, MEC13.3) to the sort scheme to purify CD45- CD31- EpCAM- mesenchymal cells. Staining was performed for 45 minutes at 4 °C, followed by a final spin-down at 550 × *g* for 5 minutes at 4 °C. Stained cells and fluorescence minus one controls were then resuspended in SB + 1:1000 DNase + 1:1000 Draq7 (Beckman Coulter) as a live/dead stain. All FACS sorting was done on a BD FACSJazz (BD Biosciences) and cells were collected in Falcon™ round-bottom polystyrene tubes (Thermo Fisher Scientific) in 300 μL DMEM + 20% CC + 2% P/S.

### Quantitative reverse transcriptase PCR

RNA was isolated from both organoids and sorted cells using a ReliaPrep™ RNA Cell Miniprep kit (Promega). The amount of RNA input for cDNA synthesis was standardized within each experiment to the RNA isolate with the lowest concentration as measured by Nanodrop (Thermo Fisher Scientific). cDNA was synthesized using iScript™ Reverse Transcription Supermix (BioRad). Primers used are as follows: *SPC*, forward 5′-ATGGACATGAGTAGCAAAGAGGT-3′, reverse 5′-CACGATGAGAAGGCGTTTGAG-3′; *Krt5*, forward 5′-TCCAGTGTGTCCTTCCGAAGT-3′, reverse 5′-TGCCTCCGCCAGAACTGTA-3′; *Scgb3a2*, forward 5′-CCACTGCCCTTCTCATCAACC-3′, reverse 5′-TGTCGTCCAAAGGTACAGGTA-3′; *L19*, forward 5′-ATGTATCACAGCCTGTACCTG-3′, reverse 5′-TTCTTGGTCTCTTCCTCCTTG-3′. Expression of each gene is relative to expression of L19 within that sample. qPCR was run on an Applied Biosystems QuantStudio 6 Real-Time PCR System (Thermo Fisher Scientific).

### Statistics

All statistical tests were performed using GraphPad Prism 7. *P*-values were calculated using unpaired two-tailed *t*-tests with Welch’s correction or ordinary one-way or two-way analysis of variance for comparisons involving multiple variables. Comparison of slopes from linear regression was calculated using an equivalent test to analysis of covariance in Chapter 18 of ref. ^40^

### Reporting summary

Further information on research design is available in the Nature Research Reporting Summary linked to this article.

## Supplementary information

Supplemental Figures 1 - 6

Reporting Summary

## Data Availability

The data that support the findings of this study are available from the corresponding author upon reasonable request.

## Supplementary information

**Supplementary information** accompanies the paper on the *npj Regenerative Medicine* website (10.1038/s41536-019-0080-9).
