# Supplementary material for: Mesenchyme-free expansion and transplantation of adult alveolar progenitor cells: steps toward cell-based regenerative therapies
Source: NPJ Regen Med. 2019 Aug 20;4:17. doi: 10.1038/s41536-019-0080-9 (PMC6702233; doi:10.1038/s41536-019-0080-9)
Supplement: Supplementary file 1 — Supplemental Figures 1 - 6 [file 41536_2019_80_MOESM1_ESM.pdf]

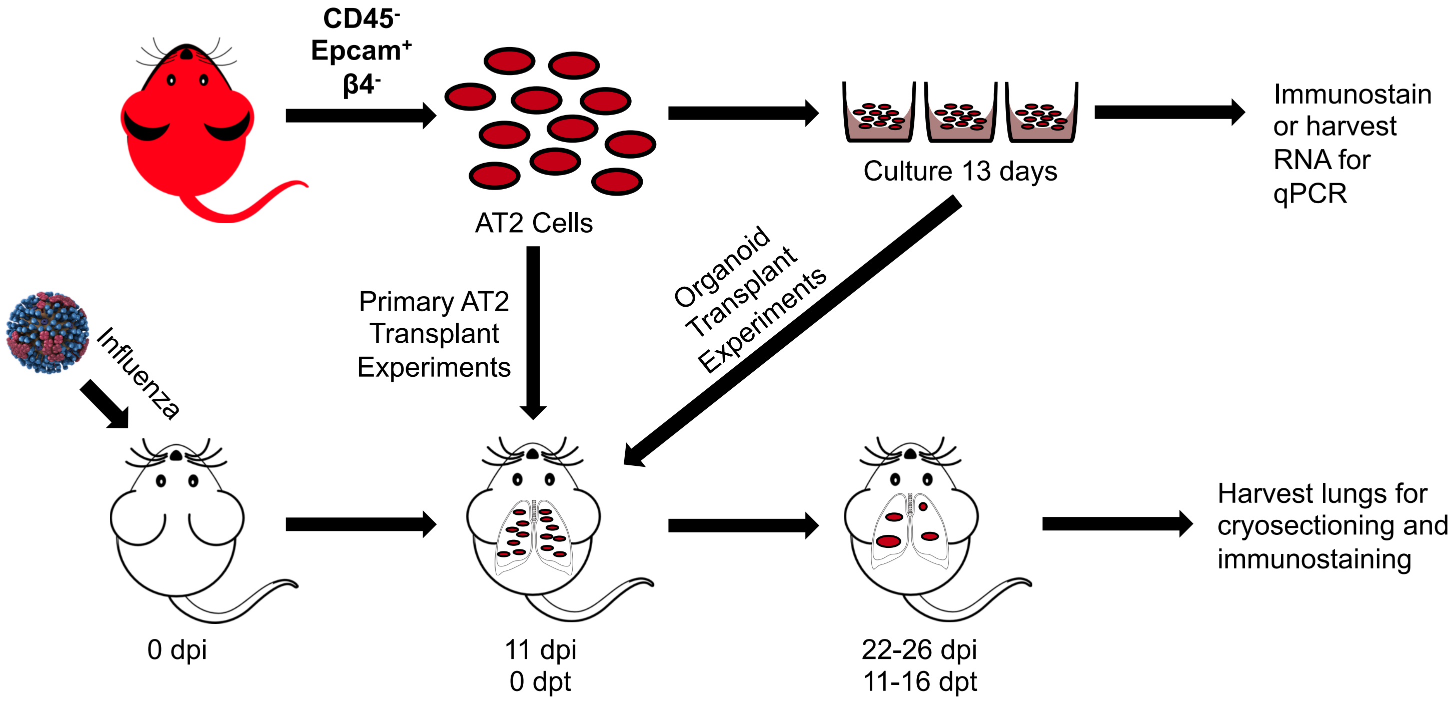

Supplementary Figure 1. Schematic of timeline and methodology for AT2 organoid culture, AT2 organoid transplant, and primary AT2 transplant experiments.

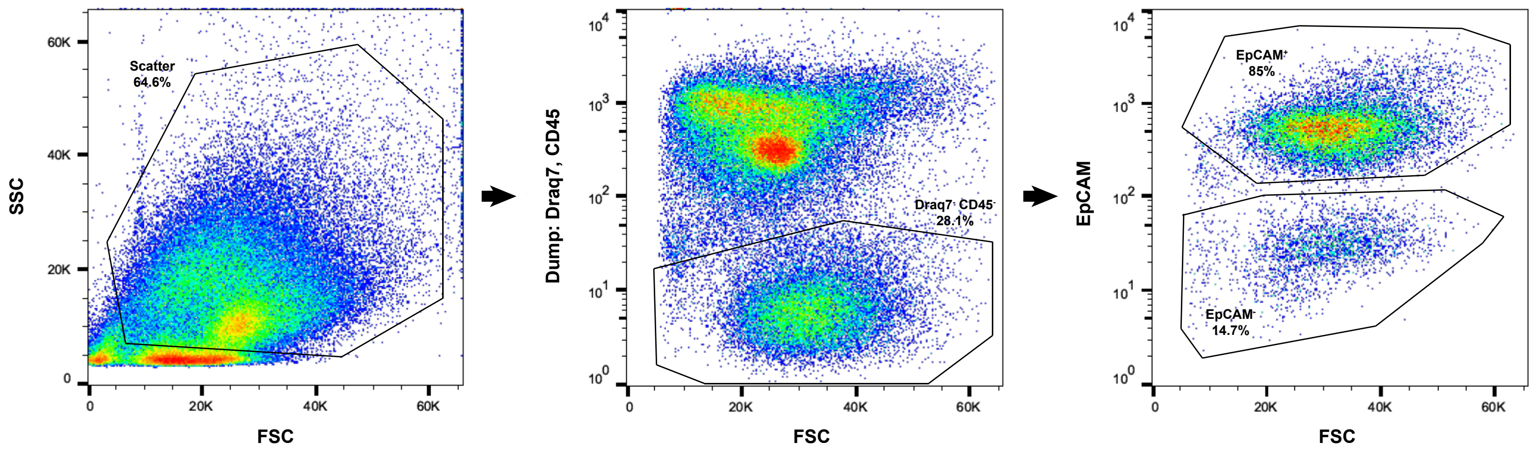

Supplementary Figure 2. Representative AT2 FACS isolation strategy up to β4 subgating.

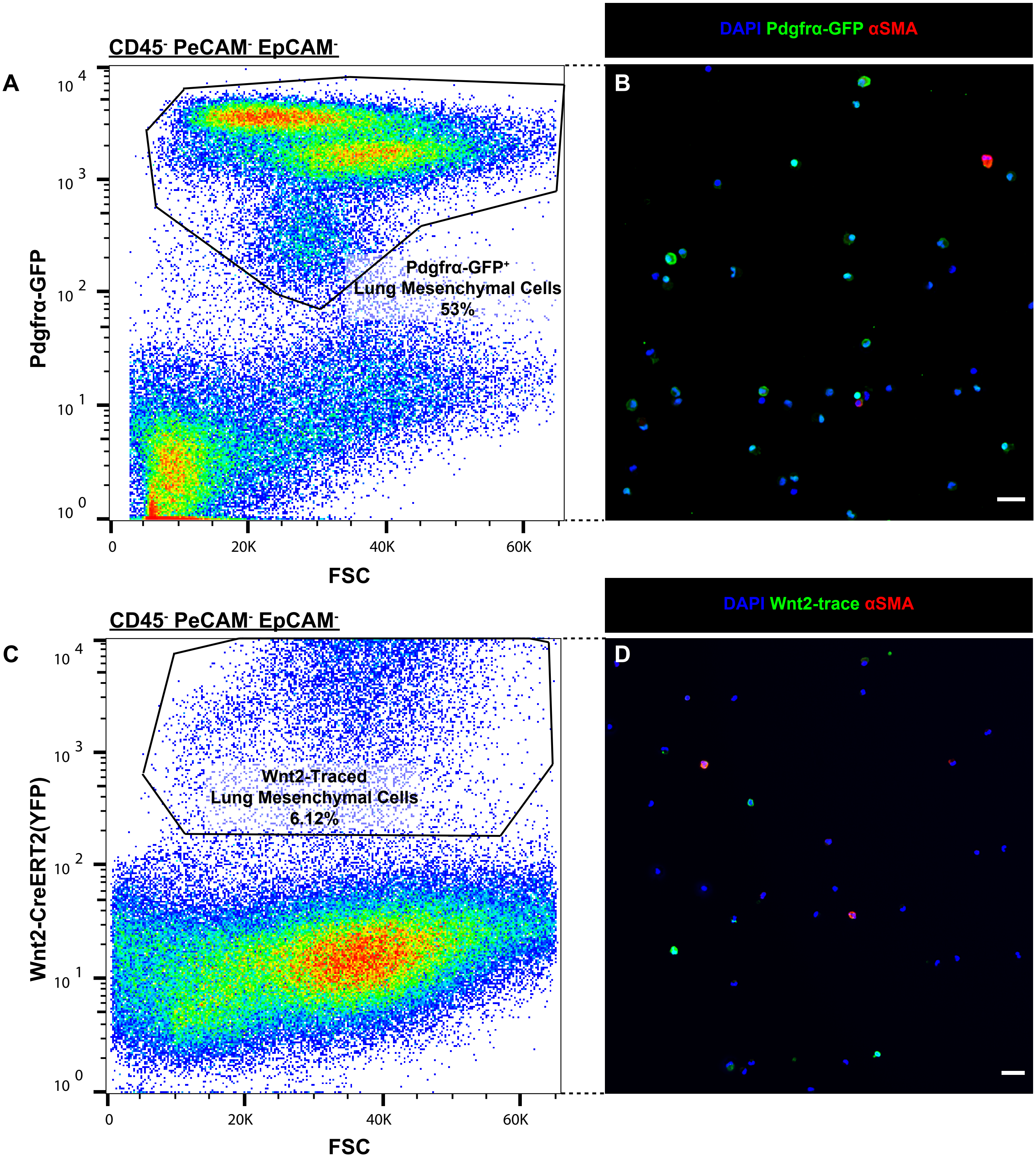

Supplementary Figure 3. Characterization of the lung mesenchyme population used for AT2 organoid culture experiments. Lung mesenchyme was obtained by FACS sorting CD45$^-$ PeCAM$^-$ EpCAM$^-$ cells from whole-lung isolate. (A, B) MANC-enriched cells comprise ~53% of the lung mesenchyme sorted using this gating scheme, as assessed by FACS (A) and cytospins (B) of lung mesenchyme from *Pdgfrα-GFP* reporter mice. Scale bar = 25μm. (C, D) The lung mesenchyme also consists of a small (~6%) subpopulation of Wnt2-trace$^+$ cells, as assessed by FACS (C) and cytospins (D) of lung mesenchyme from *Wnt2-CreERT2(YFP)* mice. Scale bar = 25μm. (B, D) αSMA$^+$ smooth muscle cells and/or myofibroblasts also make up a small proportion (~4%) of sorted lung mesenchyme.

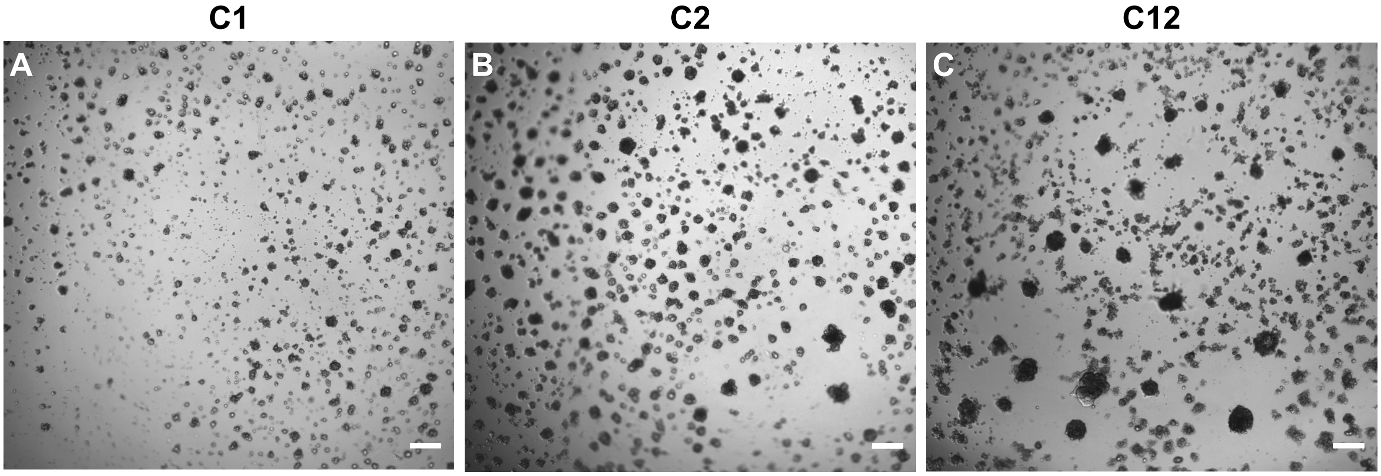

Supplementary Figure 4. FACS isolated AT2s form spherical 3D organoids in culture.
(A – C) Culture of AT2s in C1 (A) yielded smaller organoids compared to C2 (B) and C12 (C). Scale bars = 200μm.

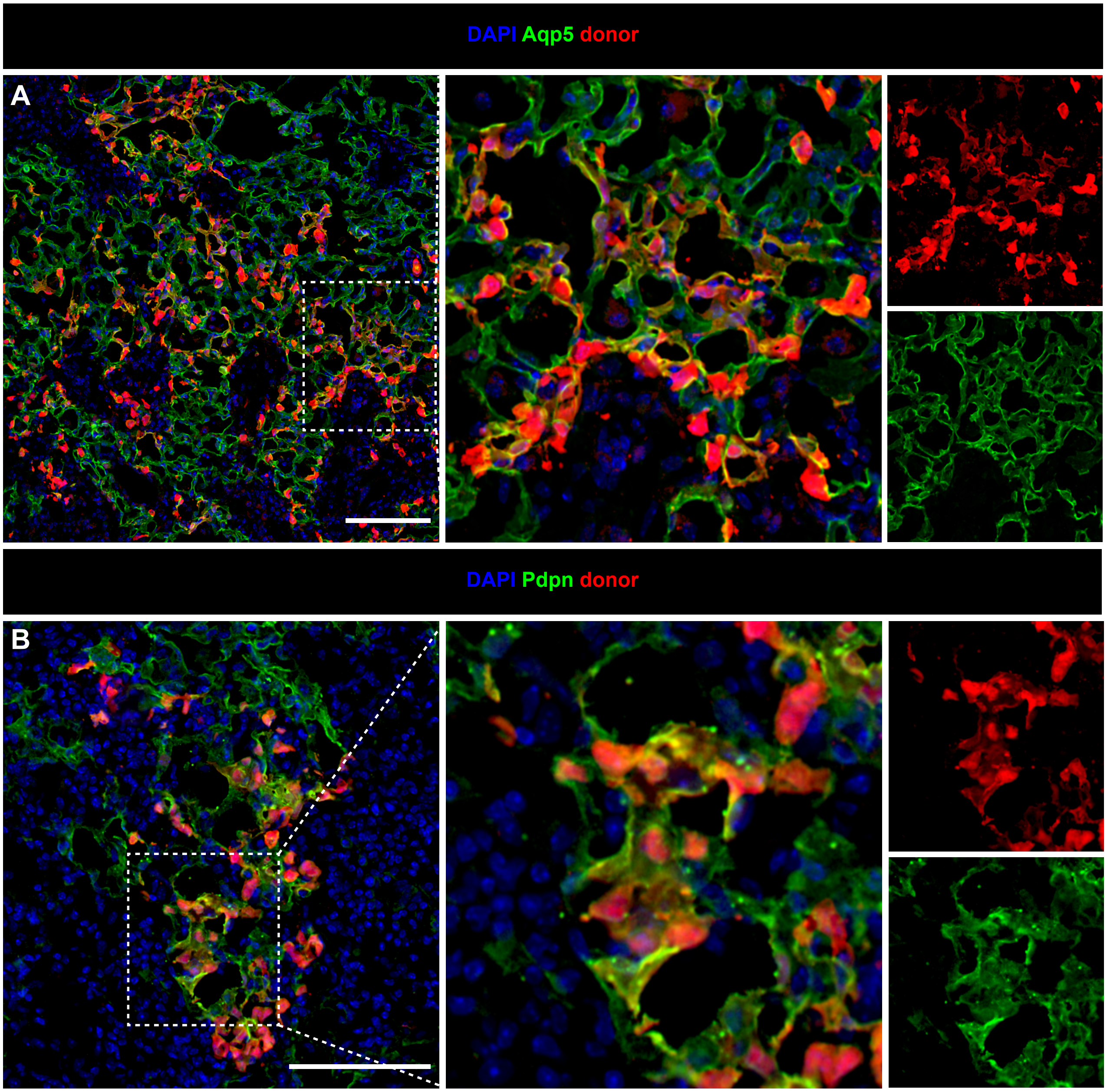

Supplementary Figure 5. Primary AT2 transplant engraftments differentiate into AT1 cells. Transplanted primary AT2 cells express additional markers of AT1 cells 13 days post-transplant such as Aqp5 (A) and Pdpn (B). Magnified sections and individual channels are derived from dashed boxes to highlight AT1 marker expression in donor cells. Scale bars = 100µm.

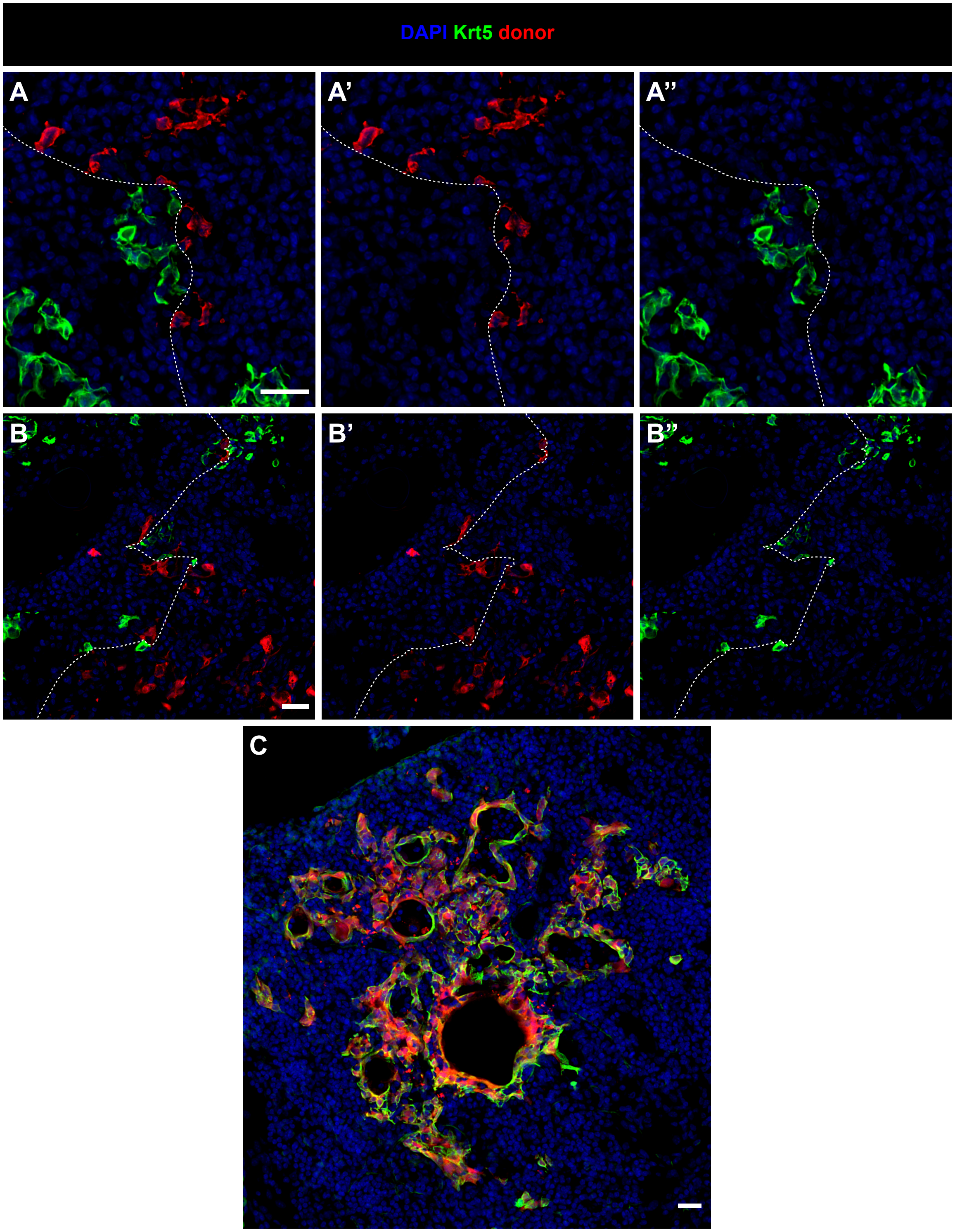

Supplementary Figure 6. Primary AT2 transplant engraftments never express markers of dysplastic regeneration. Transplanted primary AT2 cells occasionally engraft near or within areas of dysplasia, sometimes coming into contact with dysplastic Krt5+ cells yet never expressing this marker themselves (A – A'', B – B''). This is in stark contrast to transplanted AT2 organoid cells (C), which have the capacity to turn dysplastic in vivo upon transplant. The immunostains shown in (A) and (B) come from two separate primary AT2 recipient mouse lungs. Dotted lines delineate the border between primary AT2 engraftments and dysplastic Krt5+ cells. The immunostain shown in (C) comes from a separate AT2 organoid recipient mouse from the images shown in Figure 1. Scale bars = 25μm.
